# Graft-versus-host disease and impact on relapse in myelofibrosis undergoing hematopoietic stem cell transplantation

**DOI:** 10.1038/s41409-024-02220-7

**Published:** 2024-02-06

**Authors:** Sofia Oechsler, Nico Gagelmann, Christine Wolschke, Dietlinde Janson, Anita Badbaran, Evgeny Klyuchnikov, Radwan Massoud, Kristin Rathje, Johanna Richter, Mathias Schäfersküpper, Christian Niederwieser, Ameya Kunte, Silke Heidenreich, Francis Ayuk, Nicolaus Kröger

**Affiliations:** University Medical Center Hamburg-Eppendorf, Hamburg, Germany

**Keywords:** Translational research, Myeloproliferative disease

## Abstract

Allogeneic hematopoietic stem cell transplantation (alloHSCT) remains the only curative treatment for myelofibrosis (MF). Relapse occurs in 10–30% and remains a major factor for dismal outcomes. Previous work suggested that graft-versus-host disease (GVHD) might be associated with risk of relapse. This study included 341 patients undergoing their first (*n* = 308) or second (*n* = 33) alloHSCT. Anti-T-lymphocyte or antithymocyte globulin was used for GVHD prophylaxis in almost all patients. Median time to neutrophile and platelet engraftment was 13 days and 19 days, respectively. The cumulative incidence of acute GVHD grade II-IV was 41% (median, 31 days; range, 7–112). Grade III-IV acute GVHD was observed in 22%. The cumulative incidence of chronic GVHD was 61%. Liver was affected in 23% of acute GVHD cases and 46% of chronic GVHD cases. Severe acute GVHD was associated with high non-relapse mortality. The development of acute GVHD grade II and moderate GVHD was an independent factor for reduced risk for relapse after transplantation without increased risk for non-relapse mortality, while especially acute GVHD grade IV was associated with high non-relapse mortality. Last, we identified that ongoing response to ruxolitinib, accelerated-phase MF at time of transplantation and splenectomy prior to transplantation were independent predictors for relapse.

## Introduction

Myelofibrosis (MF) is a chronic myeloproliferative neoplasm characterized by clonal myeloproliferation leading to reactive bone marrow fibrosis and extramedullary hematopoiesis [1]. Clinical features include progressive anemia, hepatosplenomegaly, and constitutional symptoms. The disease occurs either de novo (primary MF, PMF) or post-polycythemia vera or post essential thrombocythemia (PPV/PET MF) [1, 2]. The introduction of molecular analyses identified three driver mutations (*JAK2, CALR* and *MPL*) and high molecular risk mutations, being associated with distinct outcomes [3–5].

Allogeneic hematopoietic stem cell transplantation (alloHSCT) remains the only potentially curative treatment option for patients with MF [6]. However, it comes with a high risk of treatment-related morbidity and mortality, and it is essential to evaluate which patients might benefit from HSCT [7]. One major factor for transplant-related morbidity and mortality is the development of graft-versus-host disease (GVHD), and patients with MF appear to develop GVHD more frequently than patients with other hematological malignancies [8].

Another major contributor to post-transplant outcomes is relapse. Although 70% of patients with PMF may achieve complete remission after alloHSCT, relapse remains a major concern, with 10–30% of patients experiencing disease recurrence within 3 years after alloHSCT [9]. Previous studies suggested disease-specific graft-versus-tumor effects with the development of GVHD and showed a significant graft-versus-MF effect associated with the appearance of GVHD [8]. More recently, Robin and colleagues observed in long-term survivors that GVHD occurring within 2 years after transplantation decreased the risk of late relapse in MF [10]. Moreover, Hernández-Boluda et al. [11] described that both acute and chronic GVHD significantly reduced the risk of relapse.

Hence, it is crucial to understand how both, acute and chronic GVHD, affect the risk of relapse, especially because large cohort studies with sufficient follow-up in MF patients allowing analysis of post-transplant events are rare [12].

This study of a homogenous cohort of MF patients was performed to dissect characteristics of GVHD after alloHSCT and to further investigate the role and significance of GVHD concerning post-transplant relapse.

## Methods

### Patients

This extensive single-center retrospective study included data from a total of 341 patients, of whom 216 (63%) were diagnosed with primary myelofibrosis (PMF), 66 (19%) with post-essential thrombocythemia myelofibrosis (post-ET MF), and 59 (17%) with post-polycythemia vera myelofibrosis (post-PV MF). Most patients were undergoing their first (*n* = 308, 90%), while 10% (*n* = 33) were undergoing their second allogeneic HSCT, which was performed at the University Medical Center Hamburg between 1994 and 2021. Conditioning regimen was busulfan-fludarabine-based for most first alloHSCT and treosulfan-fludarabine-based for most second alloHSCT [13, 14].

### Endpoints and definitions

The co-primary endpoints of the study were incidence of acute and chronic GVHD and cumulative incidence of relapse. Secondary endpoints were overall survival, non-relapse mortality, and GVHD-/relapse-free-survival (GRFS). Patients who received donor lymphocyte infusion (DLI) were censored for statistical analysis.

Chronic and acute GVHD were classified according to previously published recommendations [15, 16]. For incidence of acute GVHD, grades II-IV were used. Chronic GVHD was graded as mild, moderate, and severe according to NIH criteria. Relapse was defined either as presence of hematological or molecular. Relapse was defined according to existing criteria [6, 17, 18], such as progressive splenomegaly, loss of complete response, morphological relapse (increasing bone marrow fibrosis, increase in age-adjusted cellularity and abnormal M:E ratio, megakaryocytic abnormalities typical of MF such as pleomorphism, hyperchromasia, cloud-like nuclei and megakaryocytic clusters), decrease in conventional donor chimerism, worsening anemia, or molecular relapse [19–21]. The composite endpoint of GRFS was defined as the first event among grades III to IV acute GVHD, severe GVHD, relapse or death from any cause [22, 23].

### Statistical analysis

Descriptive statistics for continuous variables were done with Mann-Whitney-test. Categorical variables were compared using the Chi-squared method. Kaplan-Meier estimates were used for calculating survival probabilities, while probabilities of non-relapse mortality, relapse and incidence of acute and chronic GVHD were assessed using the cumulative incidence function, taking competing risks into account. For the development of GVHD, events of relapse and death without relapse were competing events. For outcome of relapse, death without relapse was the competing event; and for non-relapse mortality, relapse was the competing event. Cause-specific hazards were calculated for univariate effects on GVHD and relapse. Time-dependent modeling was created to evaluate the role of GVHD on outcomes, adjusting for potential confounders detected in univariate analysis. For multivariate survival analysis, Cox modeling was applied; and for competing risks, the model of Fine and Gray was used. All analyses were done with R statistical software version 4.0.5.

## Results

### Patients

The median age at the time of HSCT was 60 years (range, 29–75 years). Fifty-seven percent were male (*n* = 193), and 43% were female (*n* = 148). Sixty-six percent of patients (*n* = 225) tested positive for *JAK2*, 20% (*n* = 67) for *CALR*, and 4% (*n* = 15) for *MPL*. Mean variant allele frequency was 31%, 37%, and 52%, respectively. Seventy-six patients (22%) showed a mutation in *ASXL1* at the time of transplantation.

Disease risk according to DIPSS was low in <1%, intermediate-1 in 20%, intermediate-2 in 57%, and high in 21%. Forty-nine percent of patients were treated with ruxolitinib of whom 71% experienced ongoing response at time of transplantation. However, its use was discontinued at the beginning of conditioning. Nineteen patients (6%) underwent splenectomy prior to alloHSCT.

Conditioning regimes were of reduced intensity (RIC) for most patients (*n* = 314, 92%); most commonly with busulfan and fludarabine (BuFlu) (*n* = 245, 72%). Other conditioning regimens included treosulfan and fludarabine (TreoFlu) (*n* = 40, 12%) and FLAMSA-based conditioning (*n* = 44, 13%). Almost all patients (*n* = 328, 96%) received ATG for GVHD prophylaxis and a combination of ciclosporin A (CsA) and mycophenolate mofetil post-transplant. The majority of patients were treated with alloHSCT from a matched unrelated donor (*n* = 195, 57%). Median time to neutrophil and platelet engraftment was 13 days (range, 7–37 days) and 19 days (range, 4–198 days), respectively. The remaining characteristics of the total cohort are listed in Table 1.

**Table 1:** Patient and transplant characteristics.

Characteristic	Total cohort (*n* = 341)
Age in years, median (range)	60 (29–75%)
Female	148 (43%)
Myelofibrosis type	
PMF	216 (63%)
PET MF	66 (19%)
PPV MF	59 (17%)
DIPSS	
Low	2 (<1%)
Int-1	68 (20%)
Int-2	193 (57%)
High	71 (21%)
Unknown	7 (2%)
Driver mutation	
* CALR*	67 (20%)
* JAK2*	225 (66%)
* MPL*	15 (4%)
Triple negative	24 (10%)
*ASXL1*	76 (22%)
Splenectomy before transplant	19 (6%)
Ruxolitinib before transplant	
Yes	167 (49%)
No	138 (40%)
Unknown	36 (11%)
Response to ruxolitinib	
Ongoing	119 (71%)
No or lost response	48 (29%)
Reduced intensity conditioning	314 (92%)
Conditioning regimen	
BuFlu	245 (72%)
TreoFlu	40 (12%)
FLAMSA-based	44 (13%)
Other	12 (3%)
Type of HCT	
First	308 (90%)
Second	33 (10%)
ATG	328 (96%)
Donor relation	
MRD	62 (18%)
MUD	195 (57%)
MMRD	1 (<1%)
MMUD	83 (24%)
Time to first HCT in months, median (range)	61 (2–397)
Neutrophil engraftment	329 (97%)
Neutrophile engraftment in days, median (range)	13 (7–37)
Platelet engraftment	303 (88%)
Platelet engraftment in days, median (range)	19 (4–198)

### Acute and chronic GvHD

The cumulative incidence of acute GVHD was 41% (95% CI, 36–46%), which occurred after a median of 31 days (range, 7–212 days). Seventy-four patients (22%) experienced grade III-IV and 22 (7%) grade IV acute GVHD. Affected organs included skin (39%), liver (23%), and lower gastrointestinal tract (38%).

**Fig. 1:**
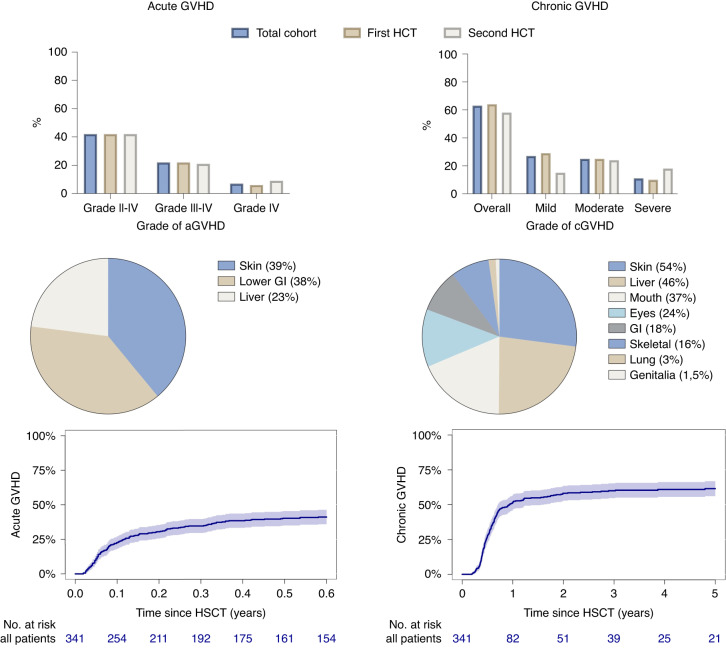
Cumulative incidence and characteristics of acute and chronic GVHD. Upper row: Distribution of different grades of acute and chronic GVHD for the total cohort and for first and second transplantation. Centre row: Affected organs in acute and chronic GVHD. Bottom row: Cumulative incidence of acute and chronic GVHD.

The cumulative incidence of chronic GVHD was 61% (95% CI, 56–67%), which occurred after a median of 198 days (range, 70–2330 days). Ninety-three patients (27%) suffered from mild, 86 (25%) from moderate and 36 (11%) from severe chronic GVHD. Affected organs included skin (54%), liver (46%), gastrointestinal tract (18%), mouth (37%), lung (3%), eyes (24%), skeletal (16%) and genitalia (1,5%). Results of acute and chronic GVHD are depicted in Fig. 1. In general, results of GVHD appeared to be stable over time, while slight decrease of acute GVHD rates were seen more recently (Supplementary Table 3).

### Outcomes

**Fig. 2:**
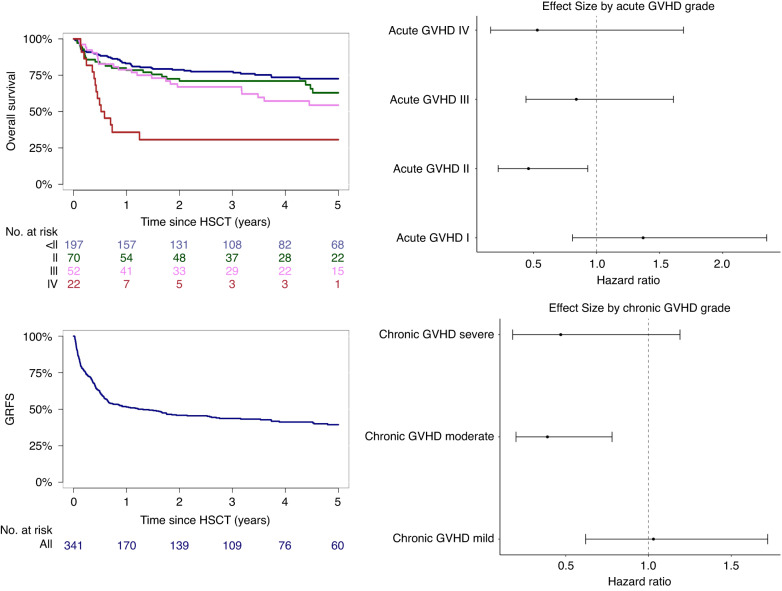
Relapse related outcomes in regard to GVHD. Upper left corner: Overall survival according to grade of acute GVHD. Bottom left corner: Overall GRFS. Right side: Hazards for relapse/progression according to grade of acute and chronic GVHD.

With a median follow-up of 5 years for the total cohort, the 5-year cumulative incidence of relapse was 21% (95% CI, 16–26%) and early relapse 1 year after transplant was 11% (95% CI, 8–15%). Overall survival was 65% (95% CI, 60–70%). Early non-relapse mortality after 1 year was 17% (95% CI, 13-21%). The 5-year composite outcome of GVHD-/relapse-free survival was 39% (95% CI, 33–45%) (Fig. 2).

Development of acute GVHD and chronic GVHD were associated with reduced risk of relapse (*p* = 0.003 and *p* < 0.001, respectively). In detail, this effect was influenced by GVHD severity (Fig. 2). While acute GVHD grade I and mild chronic GVHD did not seem to be associated with relapse in comparison with patients who did not develop GVHD, patients with grade II acute GVHD and moderate chronic GVHD showed reduced risk for relapse. Severe acute GVHD grade III-IV and severe chronic GVHD were not significantly associated with relapse.

In contrast, severe acute GVHD (especially grade IV) was also associated with higher risk for death without relapse (*p* < 0.001), showing hazard ratios (with no acute GVHD as reference) of 1.41 (95% CI, 0.70–2.83) for grade II, 1.31 (95% CI, 0.61–2.79) for grade III, and 4.32 (95% CI, 2.18–8.59) for grade IV. Corresponding 1-year cumulative incidence of non-relapse mortality was 14% (95% CI, 8–20%) for patients without acute GVHD, 20% (95% CI, 11–29%) for grade II, 19% (95% CI, 8–30%) for grade III, and 55% (95% CI, 35–75%) for grade IV. For chronic GVHD, hazard for death without relapse (with no chronic GVHD as reference) was 1.33 (95% CI, 0.61–2.89) for mild, 0.88 (95% CI, 0.33–2.34) for moderate, and 1.35 (95% CI, 0.43–4.21) for severe chronic GVHD.

### Other factors influencing GVHD and relapse

Potential risk factors for the occurrence of acute and chronic GVHD have been analyzed. Importantly, no specific influence on the occurrence of acute or chronic GVHD was identified for any given factor.

**Fig. 3:**
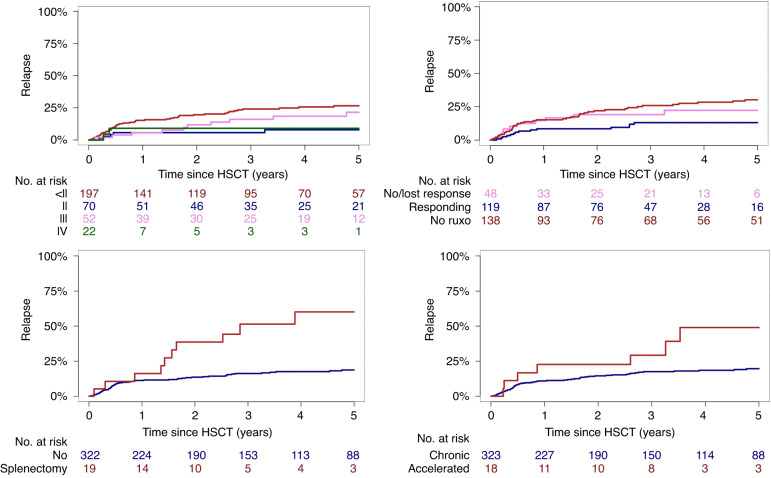
Cumulative incidences of relapse. Upper left corner: Cumulative incidence of relapse according to acute GVHD. Upper right corner: Cumulative incidence of relapse according to ruxolitinib pretreatment. Bottom left corner: Cumulative incidence of relapse according to splenectomy before transplantation. Bottom right corner: Cumulative incidence of relapse according to type of myelofibrosis (chronic and accelerated-phase).

In terms of relapse, univariate analysis showed that the type of disease appeared to influence outcome, showing higher rates of relapse for PPV MF (HR, 1.73; 95% CI, 1.01–2.97; *p* = 0.05). Furthermore, splenectomy prior to transplant was significantly associated with higher risk of relapse (*p* = 0.001), and the HR was 3.43 (95% CI, 1.91–6.17). Notably, ongoing response to ruxolitinib at time of transplantation was associated with reduced risk for relapse (Fig. 3). The remaining results of the univariate analysis on acute/chronic GVHD and relapse are shown in Supplementary Table 1.

### Multivariate analysis

Multivariate analysis identified a significant impact of acute GVHD on relapse (*p* = 0.003), showing a 54% reduced risk for relapse, with a corresponding HR of 0.45 (95% CI, 0.28-0.76). In contrast, in a time-dependent modified model, chronic GVHD was not associated with relapse, showing a corresponding HR of 0.83 (95% CI, 0.52–1.32; *p* = 0.44). Furthermore, a significantly higher risk for relapse was identified for patients with splenectomy prior to transplant (HR, 2.54; 95% CI, 1.31–4.94; *p* = 0.006). Importantly, ongoing response to ruxolitinib prior to transplantation was associated with a significantly reduced risk for relapse (*p* = 0.02), with a 51% reduced risk for relapse. The corresponding HR was 0.49 (95% CI, 0.23–0.85), whereas patients who had no response or lost their response prior to receiving transplantation showed similar risk for relapse as those who did not receive ruxolitinib. Multivariable modeling also confirmed the previously identified increased risk for patients with accelerated-phase MF, in line with previous reports (Supplementary Table 2), while disease risk according to DIPSS was not associated with post-transplant relapse [24, 25].

The only independent predictor of improved GRFS was ongoing response to ruxolitinib at time of transplantation (*p* = 0.02), with a 40% reduced risk for GVHD or relapse. The HR was 0.60 (95% CI, 0.39–0.93) in a model adjusted for diagnosis, splenectomy, accelerated-phase MF, driver mutation genotype, and presence of *ASXL1* mutation.

## Discussion

The development of GVHD after alloHSCT remains a major factor in counseling patients with MF towards curative treatment, as it usually is associated with significant morbidity. Therefore, with the present study, we aimed to investigate the characteristics of GVHD in MF and its impact on relapse in a homogenous cohort of patients. First, we found a cumulative incidence of 41% for acute GVHD at 6 months and of 61% for chronic GVHD at 5 years posttransplant. Manifestation of the liver occurred in 23% of acute GVHD cases and 46% of chronic GVHD cases. Second, our analysis showed that the development of acute GVHD grade II and moderate chronic GVHD was an independent factor for reduced risk for relapse, without increased risk for non-relapse mortality after transplantation, while acute GHVD grade IV was associated with exceptionally high mortality. Third, we identified that ongoing response to ruxolitinib, accelerated-phase MF at time of transplantation, and splenectomy before transplantation were further independent predictors for relapse.

Specific evaluation of GVHD manifestations showed higher incidence of liver GVHD (23% acute and 46% chronic) in this cohort of MF, as compared with other diseases [25]. Liver involvement usually presents in patients with signs of cutaneous and/or gastrointestinal acute GVHD, and overall incidences of acute and chronic GVHD of the liver in acute leukemia patients appeared to be 10% and 15%, respectively. Rarely (in ~5%), patients have moderate to severe hepatic GVHD without evidence of other organ involvement [26, 27]. In our MF cohort, overall incidence thus seems to be significantly higher and liver-only manifestation of acute GVHD II-IV was present in 26% of all patients with acute liver GVHD. Hepatic involvement is manifested by abnormal liver function tests, with the earliest and most common finding being a rise in the serum levels of conjugated bilirubin and alkaline phosphatase. A significant confounding factor in this regard may be the use of ATLG which is associated with hepatotoxicity. However, hepatotoxicity is usually seen early after transplantation and often reversible and studies of ATLG in acute leukemia showed significantly lower rates of liver GVHD compared with our MF cohort [28, 29]. One possible explanation for the relatively frequent occurrence of liver GVHD in MF may be present extramedullary hematopoiesis, even in absence of hepatomegaly [30, 31].

Considering factors affecting the occurrence of GVHD, no significant determinants were found in our study. Although previous research by the Center for International Blood and Marrow Transplant Research indicated a higher risk of acute GVHD in MF undergoing alloHSCT from unrelated donors, both matched and mismatched, compared to matched related donors [32], we did not find a significant difference between donor type for the occurrence of either acute or chronic GVHD. Our cohort received mostly ATLG-based GVHD prophylaxis, and previous studies suggested reduced rates of acute GVHD when using this approach in the matched related donor setting when compared with non-ATLG/ATG prophylaxis [33]. Furthermore, incidence of chronic GVHD seen in our MF cohort is significantly higher than reported in the original prospective studies of ATLG, showing rates of ~30% [29, 34, 35]. However, it is important to note that these studies included almost exclusively acute leukemia patients. The incidence of chronic GVHD in MF in our study is higher, but comparable to earlier publications as our rate of NRM is at least similar to previous reports. Nevertheless, comparing these studies can be difficult due to differences in sample size and data collection periods [32, 36, 37].

Relapse represents a major challenge in clinical practice after alloHSCT for MF. In the present study, we found that GVHD was associated with significantly reduced risk for relapse, and this effect was triggered by the development of acute GVHD II-IV and moderate-severe chronic GVHD, while no significant association of acute GVHD and subsequent development of chronic GVHD was observed. This finding is in line with another previous report from the EBMT in long-term survivors, finding that GVHD occurring within 2 years after transplant decreased the risk of relapse [10]. Another study from the EBMT showed the strength of the GVHD and graft-versus-tumor correlation was affected by type of the underlying hematological malignancy. Our results are in line with conclusions of that report, suggesting reduced risk for relapse for higher grade GVHD (acute and chronic, respectively) [8].

We only included a minority of patients receiving alloHSCT from mismatched related donors. There are several studies reporting results of haploidentical alloHSCT using the post-transplant cyclophosphamide strategy [38, 39]. However, although this approach may certainly offer curative treatment for patients for whom other donors may not be available (such as minorities underrepresented in current donor registries), evidence remains limited and particularly outcomes of relapse and GRFS were worse than reported in our present study and others [40, 41]. In addition, we included both first and second transplants. Overall survival was significantly worse after second transplantation, which was driven by higher non-relapse mortality while relapse rates were similar (Supplemental Fig. 1).

The role of splenectomy before alloHSCT remains controversial. Since the spleen has an important role in the pathophysiology of the disease, it was assumed that removing the spleen prior to alloHSCT would have a positive impact on outcome. Splenectomy was found to shorten the time for neutrophil and platelet engraftment, however, it had no effect on the overall posttransplant outcome [42, 43]. In fact, our cohort showed slightly earlier neutrophil engraftment in patients with splenectomy (median, 11 days; range, 7–19) compared with the rest of the patients (median, 13 days; range, 7–37). In contrast, we found, in line with other reports [42], that splenectomy was associated with increased risk for relapse, while overall survival was similar (*p* = 0.83).

An additional independent predictor of relapse found in our study was accelerated-phase MF at time of transplantation, consistent with previous multicenter reports [25]. Recent reports showed feasibility of hypomethylating agents in combination venetoclax, mostly in MF with blast phase [44]. Such approaches warrant further investigation.

The therapeutic effect of JAK-inhibition to reduce spleen size and improve constitutional symptoms and overall health status of patients with MF is the rationale for using drugs such as ruxolitinib before alloHSCT and to eventually improve outcome after transplantation. Several reports showed mixing results [45, 46], until a recent study from the EBMT registry showed significantly better outcomes for patients with ongoing response at time of transplantation, compared with those who did not receive any JAK-inhibitor pretreatment or those who had no response and lost response to JAK-inhibition [47]. These results are in line with our findings, while we expanded our analysis regarding the clinically relevant composite outcome of GRFS [22, 23], for which ongoing response to ruxolitinib was the only independent predictor for better outcomes. A potentially helpful tool for this evaluation might be the recently developed model for ruxolitinib response and overall outcomes in nontransplanted patients [48].

We did not find an independent association of mutational profile and relapse, warranting further investment of effort to determine the exact role of pre- and post-alloHSCT next-generation sequencing monitoring with respect to this specific outcome [49]. A most recent study found a significant effect of *TP53* multi-hit configuration, in terms of relapse and, more importantly, leukemic transformation after alloHSCT [24]. Absolute relapse rates appeared to be in line with previous work, showing 42% for multi-hit versus 18% for single-hit and 20% for wild-type configuration. There was no significant association between GVHD and *TP53* mutation.

We acknowledge several limitations. Although the present study included a large cohort of patients with extensive information, we cannot exclude selection bias and limiting comparability with other cohorts as access to transplant centers and timing of presentation might differ significantly from location to location. In comparison with other reports, we found comparable relapse and GVHD rates, while outcome in GRFS appeared to be better than previously reported in other multicenter and single-center cohorts [50]. We cannot fully exclude the possibility of bias in predictive variable selection due to the retrospective nature of our study. However, we applied standard methodologies and statistics to control for potential confounders and immortal time bias when analyzing impact of GVHD on other outcomes [51]. To fully understand the complex role of GVHD on relapse in MF patients and to further elaborate on potential predictors of GRFS, larger trials are needed [52]. Finally, definition of relapse in MF relies either on hematological features or can be detected by molecular monitoring of driver mutations. Of note, over most recent years, our practice of molecular monitoring mostly detects relapses at molecular stage, and in the present analysis indeed 36% of relapses were only molecular relapses, while the remaining cases were identified by both molecular and hematological definitions [19]. Despite the relatively large cohort analyzed here, we were unable to undergo sufficient subgroup analysis of influences on molecular versus hematological relapses.

In conclusion, we found a moderate cumulative incidence of 41% for acute GVHD, but high incidence of chronic GVHD (61%) using a homogenous reduced intensity conditioning ATLG-based approach. The development of acute GVHD grade II and moderate GVHD was an independent factor for reduced risk for relapse after transplantation without increased risk for non-relapse mortality, while especially acute GVHD grade IV was associated high non-relapse mortality. Last, we identified that ongoing response to ruxolitinib, accelerated-phase MF at time of transplantation and splenectomy prior to transplantation were independent predictors for relapse.

### Supplementary information

Supplement

## Data Availability

Data can be requested by e-mail to the corresponding author.
