# Supplementary material for: Graft-versus-host disease and impact on relapse in myelofibrosis undergoing hematopoietic stem cell transplantation
Source: Bone Marrow Transplant. 2024 Feb 6;59(4):550–7. doi: 10.1038/s41409-024-02220-7 (PMC10994836; doi:10.1038/s41409-024-02220-7)

**Supplementary Tables for:**

**Graft-versus-host disease and impact on relapse in myelofibrosis undergoing hematopoietic stem cell transplantation**

Sofia Oechsler*, Nico Gagelmann*, Christine Wolschke, Dietlinde Janson, Anita Badbaran, Evgeny Klyuchnikov, Radwan Massoud, Kristin Rathje, Johanna Richter, Mathias Schäfersküpper, Christian Niederwieser, Ameya Kunte, Silke Heidenreich, Francis Ayuk, Nicolaus Kröger

University Medical Center Hamburg-Eppendorf, Hamburg, Germany

*contributed equally

**Supplementary Table 1. Factors associated with GVHD and relapse.**

| **Factor** | **Acute GVHD** | | **Chronic GVHD** | | **Relapse** | |
| --- | --- | --- | --- | --- | --- | --- |
|  | **HR,**  **95% CI** | **P** | **HR,**  **95% CI** | **P** | **HR,**  **95% CI** | **P** |
| **Conditioning** |  |  |  |  |  |  |
| BuFlu | Reference |  | Reference |  | Reference |  |
| TreoFlu | 1.15, 0.66-2.00 | 0.62 | 1.00, 0.67-1.52 | 0.97 | 1.40, 0.75-2.60 | 0.29 |
| FLAMSA | 1.97, 0.80-4.89 | 0.14 | 0.95, 0.61-1.47 | 0.82 | 1.95, 1.09-3.47 | 0.02 |
| Other | 0.82, 0.46-1.45 | 0.49 | 0.79, 0.33-1.88 | 0.59 | 0.89, 0.22-3.51 | 0.86 |
| **Donor relation** |  |  |  |  |  |  |
| MRD | Reference |  | Reference |  | Reference |  |
| MUD | 1.15, 0.76-1.72 | 0.51 | 0.78, 0.55-1.12 | 0.17 | 0.64, 0.37-1.18 | 0.12 |
| MMUD | 1.00, 0.61-1.64 | 0.97 | 0.75, 0.49-1.14 | 0.18 | 0.75, 0.41-1.40 | 0.37 |
| **Driver mutation** |  |  |  |  |  |  |
| *CALR* | Reference |  | Reference |  | Reference |  |
| *JAK2* | 0.94, 0.62-1.43 | 0.79 | 0.78, 0.57-1.07 | 0.13 | 1.03, 0.60-1.75 | 0.93 |
| *MPL* | 2.02, 0.95-4.29 | 0.07 | 0.78, 0.41-1.49 | 0.45 | 0.28, 0.34-2.18 | 0.23 |
| Triple negative | 1.09, 0.57-2.03 | 0.79 | 0.75, 0.46-1.23 | 0.25 | 0.56, 0.21-1.51 | 0.25 |
| **HCT** |  |  |  |  |  |  |
| First | Reference |  | Reference |  | Reference |  |
| Second | 1.19, 0.67-2.16 | 0.57 | 0.86, 0.52-1.41 | 0.54 | 1.55, 0.86-2.80 | 0.15 |
| **Diagnosis** |  |  |  |  |  |  |
| PMF | Reference |  | Reference |  | Reference |  |
| PET MF | 0.80, 0.50-1.26 | 0.33 | 1.23, 0.86-1.72 | 0.21 | 1.60, 0.92-2.77 | 0.10 |
| PPV MF | 0.93, 0.60-1.43 | 0.74 | 1.16, 0.79-1.71 | 0.44 | 1.73, 1.01-2.97 | 0.05 |
| **MF grade** |  |  |  |  |  |  |
| 0-1 | Reference |  | Reference |  | Reference |  |
| 2 | 1.44, 0.64-3.25 | 0.38 | 0.97, 0.58-1.63 | 0.91 | 1.53, 0.67-3.51 | 0.31 |
| 3 | 1.46, 0.69-3.09 | 0.32 | 0.86, 0.55-1.34 | 0.50 | 0.85, 0.39-1.83 | 0.67 |
| ***ASXL1*** | 1.01, 0.68-1.52 | 0.95 | 0.76, 0.53-1.08 | 0.12 | 1.44, 0.85-2.42 | 0.17 |
| ***TP53* multi-hit** |  |  |  |  | 2.34,  1.13-5.13 | 0.04 |
| **CD34 count** | 1.00, 0.94-1.05 | 0.87 | 0.99, 0.94-1.03 | 0.59 | 0.95, 0.89-1.03 | 0.21 |
| **Blasts >9%** |  |  |  |  | 2.10,  1.43-4.79 | 0.01 |
| **DIPSS** |  |  |  |  |  |  |
| Low/int-1 | Reference |  | Reference |  | Reference |  |
| Int-2 | 0.56, 0.13-2.42 | 0.44 | 1.34, 0.19-9.69 | 0.77 | 0.82, 0.49-1.39 | 0.46 |
| High | 0.51, 0.11-2.30 | 0.38 | 1.06, 0.14-7.77 | 0.95 | 1.19, 0.65-2.16 | 0.58 |
| **Splenectomy** | 0.58, 0.23-1.46 | 0.25 | 1.16, 0.58-2.30 | 0.68 | 3.43, 1.91-6.17 | 0.001 |
| **Ruxolitinib** |  |  |  |  |  |  |
| No | Reference |  | Reference |  | Reference |  |
| No/lost response | 0.97,  0.83-1.11 | 0.72 | 0.90,  0.72-1.18 | 0.45 | 0.99,  0.84-1.16 | 0.82 |
| Ongoing response | 1.05,  0.95-1.15 | 0.30 | 1.22,  0.87-1.59 | 0.17 | 0.51, 0.32-0.82 | 0.01 |
| Acute GVHD |  |  |  |  |  |  |
| II |  |  |  |  | 0.46,  0.22-0.93 | 0.03 |
| III |  |  |  |  | 0.84,  0.44-1.61 | 0.61 |
| IV |  |  |  |  | 0.53,  0.16-1.69 | 0.28 |
| Chronic GVHD |  |  |  |  |  |  |
| Mild |  |  |  |  | 1.09,  0.60-1.73 | 0.63 |
| Moderate |  |  |  |  | 0.28,  0.07-0.74 | 0.01 |
| Severe |  |  |  |  | 0.50,  0.06-1.17 | 0.26 |

**Supplementary Table 2. Multivariable time-dependent effect of GVHD on relapse.**

|  | **Relapse** | |
| --- | --- | --- |
|  | **Hazard ratio, 95% CI** | **P** |
| **GVHD**  **(acute grade II or moderate chronic)** | 0.46, 0.28-0.76 | 0.003 |
| **Ruxolitinib** |  |  |
| No | Reference |  |
| No/lost response | 1.04, 0.82-1.20 | 0.95 |
| Ongoing response | 0.49, 0.23-0.85 | 0.02 |
| **MF type** |  |  |
| PMF | Reference |  |
| PET MF | 1.45, 0.83-2.53 | 0.19 |
| PPV MF | 1.43, 0.82-2.50 | 0.20 |
| **Splenectomy** | 2.54, 1.31-4.94 | 0.006 |
| **Accelerated-phase** | 2.52, 1.13-5.64 | 0.02 |

**Supplementary Table 3. GVHD rates over time.**

|  | **Year** | |  |  |
| --- | --- | --- | --- | --- |
|  | **2015-2021** | **2011-2014** | **<2011** | **P** |
| **Chronic GVHD** | 61% | 68% | 58% | 0.34 |
| **Acute GVHD** | 39% | 42% | 46% | 0.56 |
| II | 20% | 22% | 20% |  |
| III | 13% | 19% | 16% |  |
| IV | 6% | 2% | 10% |  |

**Supplementary Figure 1. Outcomes after first and second transplants.**


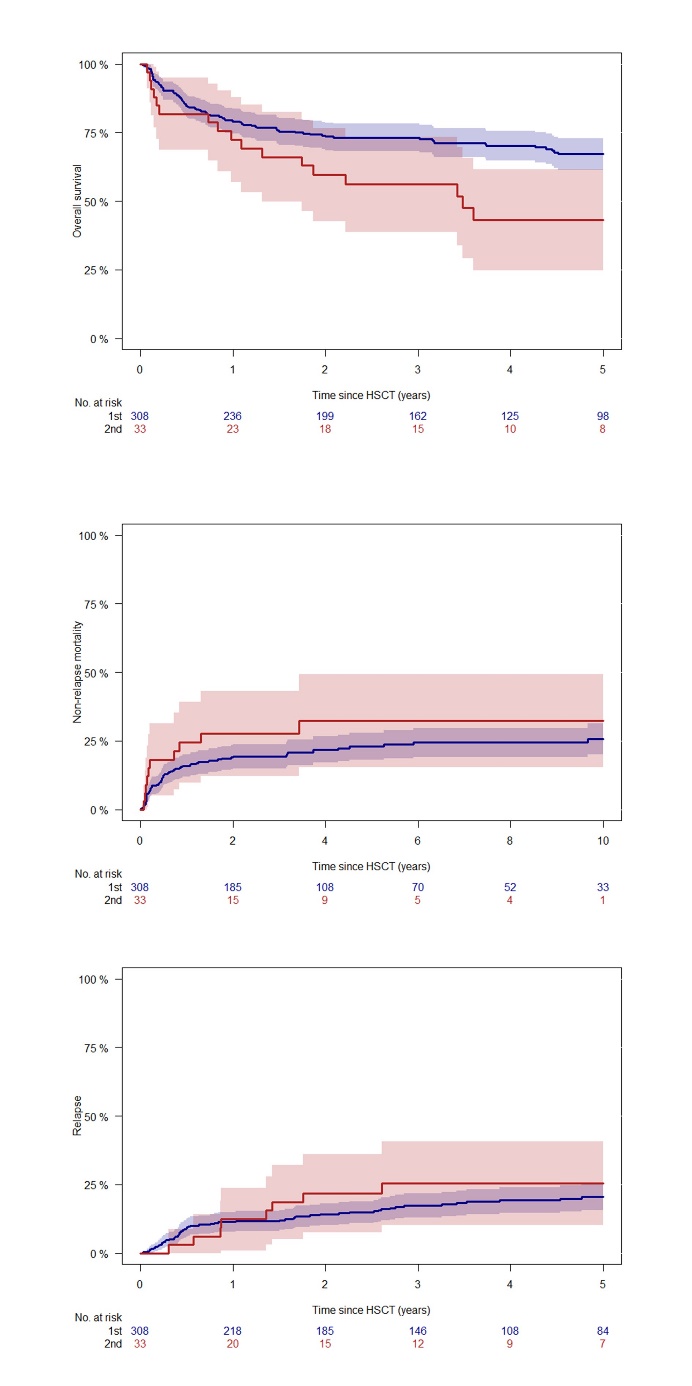

Supplement: Supplementary file 1 — Supplement [file 41409_2024_2220_MOESM1_ESM.docx]
